# Review of "Data Mining: Practical Machine Learning Tools and Techniques" by Witten and Frank

**DOI:** 10.1186/1475-925X-5-51

**Published:** 2006-09-29

**Authors:** Francisco Azuaje

**Affiliations:** 1 Computer Science Research Institute, University of Ulster, Jordanstown, Co. Antrim, BT37 0QB, Northern Ireland, UK

In the early 1990s some sectors of the computer science community were developing the idea of data understanding as a discovery-driven, systematic and iterative process. This "data mining" research and development area was expected to take advantage of the expansion and consolidation of machine learning methodologies together with the integration of traditional statistical analysis and database management strategies. The main goal was to identify relevant, interesting and potentially novel informational patterns and relationships in large data sets to support decision making and knowledge discovery. In the mid 1990s developers and users of decision-making support systems in areas such as finance (e.g. credit approval and fraud detection applications), marketing and sales analysis (e.g. shopping patterns and sales prediction) were showing a great deal of enthusiasm about the business value of data mining applications. During the next few years international conferences, journals and books were more frequently reporting advances, tools and applications in other areas such as biomedical informatics, engineering, physics, law enforcement and agriculture. Today data mining is seen as a discipline or paradigm that actively aids in the development of these and other scientific areas (e.g. Web-based computing and systems biology).

Data mining has become a fundamental research topic in the progression of computing applications in health care and biomedicine. Advances in data mining have applications and implications in areas ranging from information management in healthcare organisations, consumer health informatics, public health and epidemiology, patient care and monitoring systems, large-scale image analysis to information extraction and classification of scientific literature [1]. Approaches, techniques and applications associated with data mining has also significantly supported different data understanding and decision support tasks in bio-signal processing, such as the classification, visualisation and identification of complex relationships between diagnostic variables or groups of patients [2,3].

In "*Data Mining: Practical Machine Learning Tools and Techniques*" Witten and Frank offer users, students and researchers alike a balanced, clear introduction to concepts, techniques and tools for designing, implementing and evaluating data mining applications. Although it puts emphasis on machine learning techniques, it also introduces basic statistical and information representation methods. This book provides a variety of simple yet elegant explanations to guide the reader to understand essential concepts and approaches. The book can also be seen as a well-structured, intensive tutorial, which excels in explaining how to implement solutions to different problems.

Another reason why this book represents a significant contribution to this area is the ability of the authors to bridge gaps between conceptual and theoretical discussions, methods and practical implementations. Obviously it would not be possible (or necessary) to cover, in a single book, all the range of problems and machine learning techniques applied to different domains. However, this book also succeeds in organising and summarising significant amounts of material useful to assist the reader in justifying the selection of specific solutions. This is accomplished without making exaggerated claims or oversimplifying fundamental definitions.

The authors are also known for having led the conception and implementation of the *Weka *system. *Weka *is an open-source machine learning workbench used in this book to illustrate techniques and applications. Over the past five years Weka has facilitated educational activities at undergraduate and postgraduate levels. But also it has become a reference tool to support the assessment of machine learning technologies and their applications in biomedicine and biology [4,5].

The book is divided into two parts. The first part consists of eight chapters introducing machine learning methods, data pre-processing, model evaluation and practical implementations. An important feature is the presentation of different techniques to evaluate model predictive quality and to compare different models (e.g. cross-validation methods, probability estimations, receiver operating characteristic curves). Decision trees, different classification rule methods, instance-based learning models and Bayesian networks are some of the machine learning techniques introduced. The second part focuses on the Weka system, which offers three graphical user interfaces: the *Explorer*, the *Knowledge Flow Interface *and the *Experimenter*. In comparison to its first edition, some of the improvements include more information on neural networks and kernel models, as well as new (or updated) sections on methods, technical challenges and additional reading.

*"Data Mining: Practical Machine Learning Tools and Technique" *may become a key reference to any student, teacher or researcher interested in using, designing and deploying data mining techniques and applications. This book also deals with various aspects relevant to undergraduate or research programmes in machine learning, intelligent systems, bioinformatics and biomedical informatics.
